# How healthy is a ‘healthy economy’? Incompatibility between current pathways towards SDG3 and SDG8

**DOI:** 10.1186/s12992-019-0532-4

**Published:** 2019-12-02

**Authors:** Mariska Meurs, Lisa Seidelmann, Myria Koutsoumpa

**Affiliations:** Wemos Foundation, Ellermanstraat 15-O, 114 AK, Amsterdam, the Netherlands

**Keywords:** Malawi, Tanzania, Uganda, IMF, SDGs, Economic growth, GDP, Health expenditure

## Abstract

**Background:**

The interconnections between health and the economy are well known and well documented. The funding gap for realizing SDG3 for good health and well-being, however, remains vast. Simultaneously, economic growth, as expressed and measured in SDG8, continues to leave many people behind. In addition, international financial institutions, notably the International Monetary Fund (IMF), continue to influence the economic and social policies that countries adopt in ways that could undermine achievement of the SDGs. We examine the incoherence between the economic growth and health goals of the SDGs with reference to three East African countries, Malawi, Uganda, and Tanzania, where our organization has been working with partner organizations on SDG related policy analysis and advocacy work.

**Results:**

In all three study countries, some health indicators, notably infant and child mortality, show improvement, but other indicators are lagging behind. Underfunding of the health sector is a major cause for poor health of the population and inequities in access to health care. GDP increases (as a measure of economic growth) do not automatically translate to increases in the countries’ health spending. Health expenditure from domestic public resources remains much lower than the internationally recommended minimum of USD 86 per capita. To achieve this level of health spending from domestic resources only, GDP in these countries would require an unrealistic manifold increase. External aid is proving insufficient to close the funding gap. IMF policy advice and loan conditionality that focus on GDP growth and tight monetary and fiscal targets impair growth in health and social sector spending, while recommended taxation measures are generally regressive.

**Conclusions:**

The existence of the GDP-focused SDG8 can delay efforts towards the achievement of the SDG3 for health and well-being if governments choose to focus on GDP growth without taking sufficient measures to equally distribute wealth and invest in the social sectors, often under the influence of policies advised or conditions put in place by the IMF. Although the IMF has started to acknowledge the importance of social development, its policy advice still adheres to austerity and pro-cyclical economic development harming a country’s population health. To realize the SDGs everywhere, governments should abandon GDP growth as a policy objective and place more emphasis on SDG17 on global co-operation.

## Introduction

The Sustainable Development Goals (SDGs) of Agenda 2030, in its comprehensive set of goals and indicators, recognize the many interlinkages that exist between various aspects of well-being. However, there is insufficient acknowledgement that some of the goals – or at least the ways in which they are operationalized – contradict each other. In this article we explore this incoherence in the SDGs by focusing on three East African countries (Malawi, Uganda, and Tanzania) in which we have been working to support their efforts to improve their health outcomes. We argue that the way in which economic growth is being pursued in these three countries, as operationalized in SDG target 8.1, hampers progress on SDG3, to *‘ensure healthy lives and promote well-being for all at all ages’*. At the same time, the lack of progress on SDG17, *‘to revitalize the global partnership for sustainable development*’, impedes global equity and poverty eradication in low-income countries (LICs) as evidence shows.

## Background

After the era of the Millennium Development Goals (MDGs), the world’s leaders recognized that although progress had been made in some areas, many objectives had not been accomplished. Agenda 2030 continues the unfinished agenda of the MDGs but is more ambitious and comprehensive, acknowledging that more systemic policy changes are needed in both high- and low-income countries to successfully address ongoing health challenges facing the world’s population. Alongside concrete targets on, for instance, poverty reduction and improved health outcomes, Agenda 2030 places strong emphasis on reducing inequities and the need for fairer economic arrangements at the global level.

The call for such a comprehensive agenda is not new. Already at the Conference on Primary Health Care in Alma Ata in 1978 political leaders called for a ‘*New International Economic Order’* and emphasized that the world’s *‘sustained economic and social development’* is only within reach if its people are healthy [1]. This was also a central message in the report of the World Health Organization’s (WHO) Commission on Social Determinants of Health, which attributed persistent poverty and inequities to a *‘toxic combination of poor social policies and programmes, unfair economic arrangements and bad politics’* ([2 p. 1). The earlier WHO Commission for Macroeconomics and Health, released around the same time as the MDGs, pointed out that investments in health represent a useful and successful poverty reduction strategy, and that investments to improve population health would lead to better and stronger economic growth [3].

More recently, in 2016, experts from the International Labour Organization (ILO), the Organization for Economic Cooperation and Development (OECD), and the WHO drew attention to how the health sector should be considered an economic resource-generating sector, not only by promoting a healthy and more productive population, but also by providing possibilities for paid employment [4]. The goal of this High-Level Commission on Health Employment and Economic Growth (UNHEEG) was to stimulate countries to create 40 million new jobs in their health and social sectors as a means for inclusive economic growth in the SDG era. The Commission report estimated that for every additional year of life expectancy a country creates through health improvements, it generates a 4% increase in GDP [4].

What does it take, then, to move from acknowledgement to action? The comprehensive nature of the Agenda 2030 represents not only an opportunity but also a challenge, as governments and multilateral organizations may use the extensive list of goals and targets as a ‘shopping list’, cherry-picking their favourites, or those easiest and less threatening to implement, rather than adhering to the Agenda in its intended holistic manner. The interlinkages between the goals are not automatically translated into a comprehensive policy-making approach, with implications for health improvement. Health remains an important part of the Agenda 2030, as reflected in the ‘health goal’ SDG3 on healthy lives and well-being for all with its expansive targets focusing on main health threats such as infections, non-communicable diseases, road accidents, and pollution, by fostering access to quality health services without inflicting financial hardship. While the accomplishment of SDG3 is an end in itself, it is also an important means to contribute to other SDGs, notably SDG8. The reverse question, however, is whether SDG8 unequivocally contributes to SDG3.

The objective of SDG8 is to promote sustained, inclusive, and sustainable economic growth, full and productive employment, and decent work for all. Its first target (8.1) is to sustain per capita annual economic growth at a level of at least 7% of the GDP for the least developed countries (LDCs), which include our three focus countries [5]. The use of the GDP as an indicator is not surprising, given that it is a widely-used indicator, is measured frequently, and allows inter-country comparisons. Moreover, there is a broad consensus among countries on the technical definition of the GDP. By extension, since its establishment in the Bretton Woods conference in 1944, the International Monetary Fund (IMF) has been using the GDP as its main tool in measuring a country’s economy, as can be seen in its prominence in the IMF’s indicators [6, 7].

However, the pursuit of a GDP target in itself does not ensure either sustainability or inclusivity. Although this is recognised in different targets under SDG8, in practice, economic policies are often focused on a few macro-economic indicators, such as consistent GDP growth, low inflation, and a balanced budget. In many LICs and lower-middle income countries (L-MICs), this focus in their economic policy goals is often driven by policy advice from the IMF, or determined by conditions tied to IMF loans [8]. It is well documented that the World Bank’s and IMF’s Structural Adjustment Programs (SAPs) in the 1980s and 1990s applied a one-size-fits-all approach targeting reductions in government spending and promoting deregulation and privatization [9–12]. In many countries, this led to reductions in public investments in health and education, the negative effects of which are still being felt [10]. Following extensive criticisms in the early 2000s, the IMF and World Bank started adopting more flexible adjustment approaches that emphasized poverty reduction. As of 2010, IMF programs also include social protection floors, aimed at increasing spending on public services such as health and education [13].

In spite of the rhetoric that things have changed [14], current policy conditionality under IMF loans still requires general fiscal austerity, posing unnecessarily tight limits on public spending [15]. Targets for budget deficits and inflation remain low, in general arbitrarily set at 3 and 5% respectively, although there remains no consensus on the necessity for such low rates. These low targets impede governments from being able to increase their social spending [16]. While the IMF now includes ‘priority’ expenditures on social programs, like distinct health programs or primary education, these pro-poor conditions are non-binding and non-compliance with them does not undermine ongoing financial support by the IMF. Research in 16 West African countries with IMF programs in the period 1995–2014, finds that less than half of social spending targets were met. In several of these countries the IMF advised against increases of social spending out of concern that these increases would not be sustainable. Moreover, health spending in this sample of 16 countries was negatively correlated with the number of binding conditions included in the program [10]. Perhaps indicative of the dominating influence of fiscal austerity, similar research in West African countries with IMF programs between 1985 and 2014 found that even when social spending floors were not met, budget balance conditions were consistently abided by and often far-exceeded [11].

In this article, we discuss how the focus on SDG8.1, and the way in which GDP growth is pursued with a focus on austerity, can impair or delay the realization of SDG3 for health and well-being for all. We express concerns on the choice of GDP as an SDG indicator of inclusive and sustainable economic growth in general, and how it may undermine the prioritization of social sectors, including health, and hamper equity. We discuss alternative indicators for, as well as alternative paths towards, sustainable development, and the need for drastic action at the global level to promote economic justice. Without this, it will not be possible to realize the Agenda 2030.

## Methods

To examine whether SDG8 and SDG3 were compatible or contradictory, we reviewed literature and data from a variety of sources. As part of the policy and context analyses on health financing and human resources for health in Malawi, Tanzania and Uganda, which is part of our organization’s program of work, we have been reviewing literature on the types of policy advice these countries receive from the IMF and their impact on health investments. Based on this body of literature on economic policies, adjustment and the impact of austerity measures, we analysed IMF policy advice in these three countries on targets for budget deficits, inflation rates, wage bill containment, and fiscal policy. The IMF country documents were retrieved from the IMF country specific webpages, and we analysed the relevant program documents and article IV consultation reports for Malawi, Tanzania and Uganda over the period 2016–2018. In addition, we searched for secondary literature on the impact of structural adjustment in these countries from the start of their engagement with the IMF. We then accessed information specific to the health systems of our three focus countries using the Global Health Expenditure Database from the WHO [17] for health expenditure data, the WHO Global Health Observatory [18], the World Bank Health Nutrition and Population Statistics database [19], and countries’ public health policy documents.

## Results

### Gaps in health and health resources

In spite of progress made since the start of the ‘MDG-era’, the health situation of many people in LICs and L-MICs remains worrisome. Improvements have been made in infant and child mortality, but maternal mortality is conspicuously lagging behind. At the High Level Political Forum (HLPF) in 2017, progress towards SDG3 was reported on by the WHO, which noted that for many indicators the inequality between income groups remains striking:

Social determinants greatly impact on child survival and death as children from the poorest households are, on average, nearly twice as likely to die before the age of five as children from the richest households as shown by survey data from some 50 countries. ([17] p. 3)

The report highlights the acceleration required to achieve the target for reducing maternal mortality: an annual reduction of at least 7.3%, which is more than triple the rate attained between 1990 and 2015. One of the main obstacles identified is the lack of skilled care, aggravated by the global shortage of health workers [20]. Hence, the report emphasized the need to create more fiscal space for expanding health sector employment and health protection, identifying underfunding as a major cause for low health status and inequities in access to health care.

Meanwhile, the funding gap to realize SDG3 remains huge. Additional resources needed to make progress towards the SDG3 targets in low- and middle-income countries (LMICs) are estimated to range between USD 274 billion and USD 371 billion per year by 2030 [21]. This is a massive gap when compared to the available domestic resources and external funds for health. After two decades of growth, the level of official Development Assistance for Health (DAH) has levelled since 2011, and slightly decreased from 2017 to 2018, reaching a total of USD 38.9 billion [22].

### Country situation

#### SDG3 progress – key health indicators and health spending

Comparing key health indicators in our focus countries with the targets set in SDG3 clearly illustrates that the remaining challenges are substantial, even if in some instances better than Sub-Saharan African averages but vastly worse than OECD averages (Table 1). Overcoming these challenges will require a huge effort and considerable investment in the health sector.

**Table 1:** Selected health indicators in focus countries compared to SDG3 targets

Health Indicator	Malawi	Tanzania	Uganda	SDG3 target	OECD average	Sub-Saharan African average
Maternal mortality per 100,000 live births (2015)	634	398	343	70	14	547
Under five mortality per 1000 live births (2017)	55	54	49	25	6.7	75.5
Neonatal mortality per 1000 live births (2017)	23	21	20	12	3.7	27.2

*Source*: World Bank Health Nutrition and Population Statistics [19]

As indicated in the SDG3 progress report, the shortage of health workers is one of the main obstacles towards improving access to health services and is caused in large part by insufficient funding [20]. Based on a SDG index threshold of 4.45 physicians, nurses, and midwives per 1000 population, the WHO has calculated that there is a global shortage of 17.4 million health workers, with the largest challenges being in the African region [23]. Although country specific calculations are needed for national planning purposes, the threshold gives an indication of the minimum number of health workers needed to realize the SDGs. The comparable numbers in our focus countries are far below this threshold, as per the most recent data available in the WHO Global Health Observatory: 0.35 for Malawi (in 2009), 0.44 for Tanzania (in 2014) and 0.75 for Uganda (2015) [18]. In Malawi, the number has gone up slightly since then, to 0.5 in 2016, but decreased for the number of nurses [24].

We compared current government spending on health in relative and absolute terms with the amount required to meet the internationally recommended levels. In our analysis, we refer to health spending targets recommended by the Working Group on Health Financing at the Chatham House Centre on Global Health Security in 2014 [25], which consisted of both a relative target (> 5% of GDP) and an absolute target (≥USD 86 per capita). Linking public expenditure for health to a country’s wealth, as reflected by the GDP, motivates governments to raise more revenue for social services and prioritize health in their budget. However, because in most LICs and L-MICs 5% of GDP will not yield sufficient levels of per capita spending, an absolute target of USD 86 per capita is used.

The absolute target of at least USD 86 per capita is based on 2014 data, which needs regular updating in line with changing price levels. More recently, the World Bank and the WHO refer to slightly higher figures of USD 90 and USD 112 per person per year to deliver an essential health benefit package [21, 26]. The earlier Working Group targets, however, were adopted by the African Union at its 2016 summit in Rwanda as benchmarks for the Africa Scorecard on Domestic Financing for Health [27] and so are the data used for our three focus countries.

**Fig. 1:**
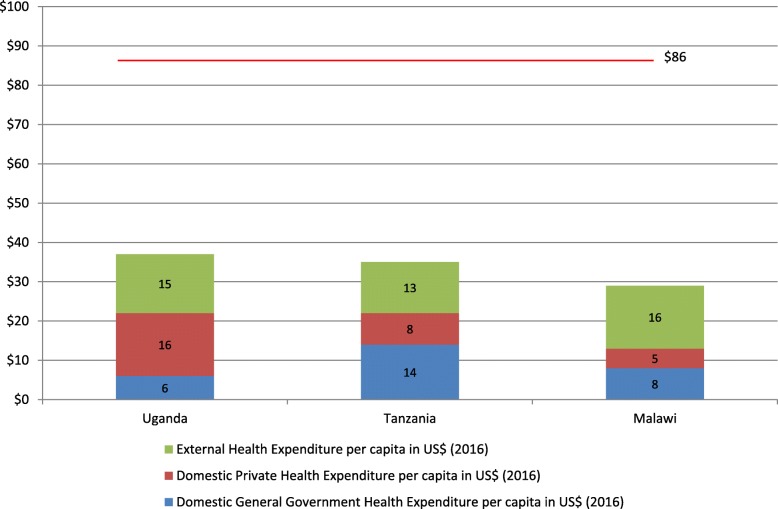
Health expenditure in USD per capita by source (2016) compared to the international minimum target

As in many LICs and L-MICs, the current total spending on health in Malawi, Uganda and Tanzania is far below the recommended level, as can be seen in Fig. 1. However, it needs to be emphasized that the target of USD 86 is the amount that should be raised from public sources alone. When looking only at the domestic general government health expenditure (GGHE-D) of USD 8, 6, and 14 in Malawi, Uganda, and Tanzania respectively [17], it is clear that this amount is insufficient to fund a basic health care benefit package. Moreover, health expenditure from external sources does not contribute sufficiently for the countries to reach the minimum target of USD 86 per capita. Notably, in Uganda, private households contribute USD 16, over twice as much as what is provided publicly. Even if these figures are lower in Malawi and Tanzania, at USD 5 and 8 respectively, they are still too high to achieve the SDG3 Universal Health Coverage (UHC) target which aims to provide quality health services to all without causing financial hardship.

**Fig. 2:**
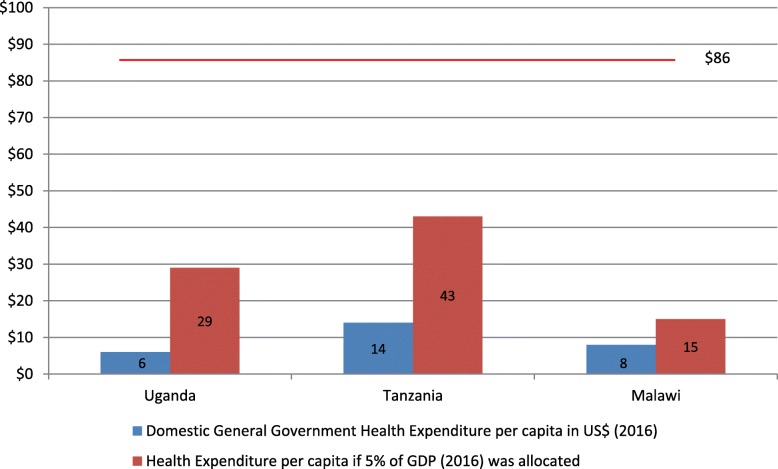
General government health expenditure in USD per capita and if 5% GDP was allocated (2016)

None of the three countries would meet the USD 86 per capita benchmark even if they met the relative target of allocating 5% of their GDP to health. As shown in Figs. 2, 5% of the GDP in 2016 would have translated only to USD 15, 29 and 43 per capita in Malawi, Uganda and Tanzania, respectively.

With their current levels of GDP, these countries’ capacity to raise sufficient domestic resources for health is limited. In Malawi, the health budget would need to increase from USD 177 million (2017/18 budget) to USD 1.5 billion to meet the recommended level of USD 86 per capita [28]. For Uganda to reach the USD 86 for the estimated population in 2019 [29], the health budget would have to increase from the approved budget of USD 335 million for 2018/19 [30, 31] to USD 3.5 billion. In Tanzania, an allocation of 5% of GDP to health would result in USD 43 per capita. While representing the highest gain of the three countries investigated, this would still fall short of the USD 86 per capita threshold. At the level of Tanzania’s population in 2017 [32], to meet this target the budget would need to increase from its present commitment of USD 742 million for 2018/19 [33] to USD 4.9 billion.

To achieve such levels of health budget only with domestic resources would require massive increases in the countries’ GDP. GDP per capita in 2016 (in current USD) was USD 301 for Malawi, USD 610 for Uganda, and USD 857 for Tanzania [17]. To achieve the minimum spending of USD 86 per capita derived from 5% GDP allocation to health exclusively from domestic resources would require at least USD 1720 per capita. This would equate to an almost 6-fold increase in Malawi, 3-fold in Uganda and double in Tanzania. Even at the best-case LDC target of 7% annual per capita GDP growth in SDG8, this would take decades to achieve.

**Fig. 3:**
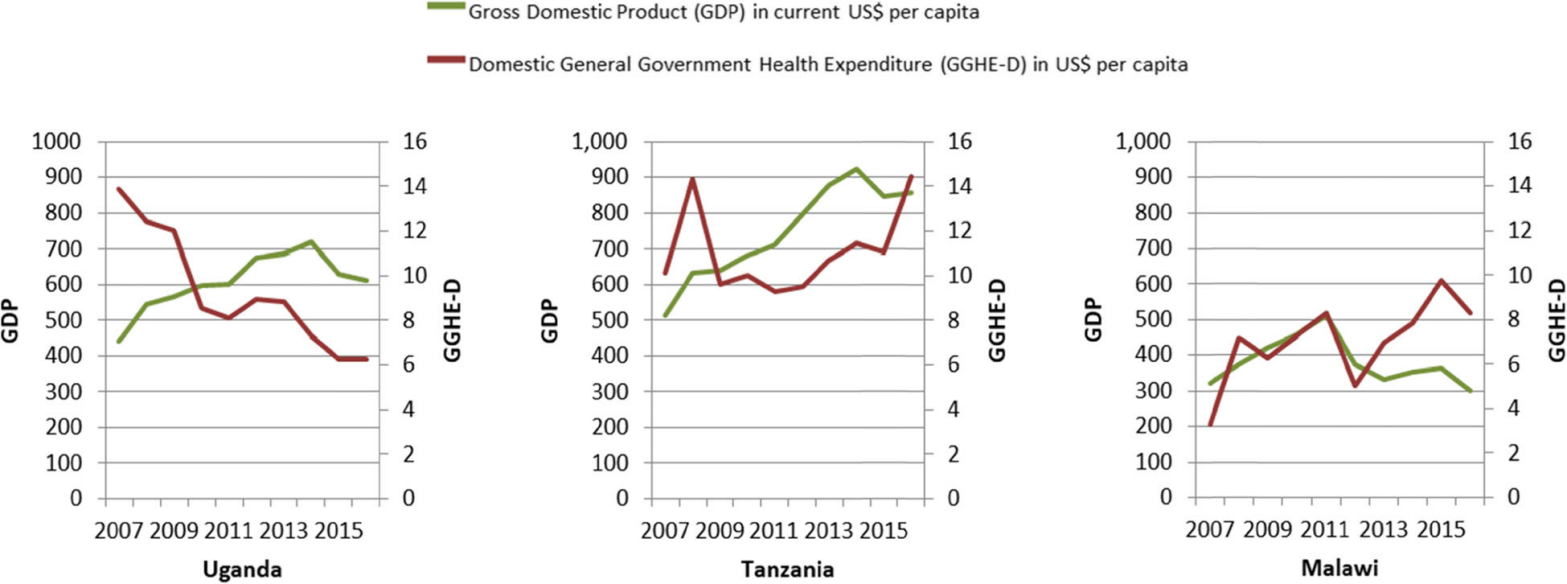
General government health expenditure juxtaposed to the GDP in USD per capita (2007–2016)

Furthermore, this GDP growth would need to translate into higher allocations to health. Exploring data for the decade 2007–2016 [17], we observed that in our focus countries, general government health per capita expenditure from domestic sources (GGHE-D per capita) has been following different trajectories compared to GDP per capita trends (Fig. 3).

The trends differ across the three countries. Tanzania saw increases in both GDP and GGHE-D per capita. In Malawi, GDP per capita decreased slightly in the period analysed, however, the GGHE-D per capita increased more than twofold. Increased GGHE-D per capita in both countries represents a health-positive, if still inadequate, improvement. In contrast, Uganda saw increases in its GDP per capita while GGHE-D per capita fell by more than half. This may be attributed to external aid, currently representing 40% of the total health expenditure [17], crowding out GGHE-D.

### IMF economic policy advice and conditionality in Malawi, Tanzania and Uganda

We conducted a qualitative analysis of recent IMF programs for our three focus countries, based on official IMF program documents. In our analysis, we screened for the main ways in which these programs influence health spending: targets to reduce the budget deficit (via measures to reduce or contain public spending, including public employment reduction, and/or increases in taxation) and tight monetary policy.

### Malawi

In Malawi, SAPs have been implemented since 1981, but these have not resolved the country’s indebtedness or set it on a path of economic growth. According to a World Bank analysis, real GDP per capita growth was only 1.5% between 1995 and 2015 with little impact on poverty; poverty remained stagnant at more than 50% and actually increased in rural areas between 2011 and 2014 [34]. Comparing to the period preceding adjustment, the economy has worsened on many accounts: per capita income decreased by 0.7% during the adjustment period, the average annual inflation rate more than doubled (from 8.4 to 22%) and while export slightly increased, it did not become more diversified [35].

An analysis of Malawi’s current program with the IMF – a three year Extended Credit Facility (ECF) approved in April 2018 – and its first review in November 2018, reveal that both fiscal tightening and inflation targeting are prominent parts of the program [36, 37]. Policy measures to cut back expenditures include reducing the budget for maize procurement and agricultural subsidies, reinforcing the implementation of the automatic fuel price mechanism, increasing the level of fees for certain public services (not further specified), and restrictions on the wage bill (limiting wage increases to a maximum of the inflation rate and recruitments only in essential cases). Malawi did not meet the condition on reducing its primary deficit, however, partly due to an additional 5% wage increase for government employees in the bottom half of the wage scale and hiring of new medical workers. The November 2018 review emphasizes that extra spending incurred in 2017/18 will be compensated by ‘*tightening the fiscal stance in 2018/19’* ([35] p. 9).

The program states that budget cuts will target non-essential expenditures and that social spending will be maintained, but the general description does not allow assessment of the implications on household income across the different income groups. The program aims at protecting social spending through an increase in spending for health and education of 0.4% of GDP over the program period. With current GDP, that would amount to USD 25.2 million over the three-year period, or USD 8.4 million on an annual basis, which is a small amount when compared to the funding gap for health. Malawi has increased the level of government revenue as a percentage of GDP in recent years, from 14.5% in 2009 to 17.3% in 2017 [32]. While both trends are in a potentially more health-positive direction, and the November IMF review report notes that *‘social spending allocations in the government budget will not be adjusted downward to meet fiscal targets of the program’* ([35] p. 91), they are far below the levels needed to achieve the recommended minimum health spending benchmarks.

To increase government revenue while meeting fiscal targets, the IMF program advises extending the coverage of Value Added Tax (VAT) and reversing the VAT exemption on cooking oil and other ‘unnecessary exemptions’. Consumption taxes such as VAT are generally regressive and disproportionately affect the poor. More progressively, the IMF program also recommends strengthening the capacity of the revenue authority, repealing the industrial rebate scheme, and discontinuing the granting of tax holidays.

Tight monetary policy is a key objective stressed in the ECF program; the government aims to lower inflation to 5% in the medium term. Between the end of 2017 and August 2019, inflation rose from 7.1 to 9.3% and is expected to increase further due to higher prices for maize, electricity and fuel. In response to inflationary pressures, the Reserve Bank of Malawi maintained its policy rate (the rate at which the central bank lends to other banks) at 16% and the government has expressed its commitment to adopt inflation-targeting over the medium-term.

### Uganda

Uganda became a member of the IMF back in 1963 and in 1987 obtained an IMF loan under the Structural Adjustment Facility, which was extended in the periods 1989–1992 and 1992–1997 [12]. Based on the conditionality of this loan, Uganda had to liberalize its economy. As a result, the fixed foreign exchange policy changed to a floating system, and in order to control inflation, the Uganda Shilling was devaluated. In the period of SAPs, many public servants were discharged in order to reduce the government wage bill, trade unions were weakened, and the cooperative movement started to crumble [38]. Regarding taxation policies, historically, most of the tax revenue derived from customs and excise on international trade. This changed in the early 1990s, when the IMF promoted reforms to reduce tariffs on international trade and increase income tax collection, along with the introduction of the VAT.

Uganda is currently under the Policy Support Instrument (PSI), an IMF tool that enables LICs to receive advice and support from the IMF without a borrowing arrangement. The PSI helps countries to design what the IMF considers to be effective economic programs, and thereby delivers a clear signal to donors, multilateral development banks, and international financial markets of an IMF endorsement of the strength of a member country’s policies and credibility [39].

In a review by the IMF in 2017, the IMF complimented Uganda for bringing inflation down to 5%. This inflation-targeting framework was introduced in 2011 and replaced the monetary-targeting framework. The review still recommended the Bank of Uganda to further tighten monetary policy if drought-induced increases of food prizes drove up inflation [40]. Uganda’s GDP per capita has been growing steadily in the last decade [32]. However, the Government captured only 14.6% of the GDP through taxation in 2018, a percentage that has increased by 3% since 2011 but which is still rather low [41].The IMF recognized that the 2% GDP health spending is rather low, in fact lower than the East African Community average, but advised the authorities to consider increasing social spending only once economic growth has recovered [40].

### Tanzania

Tanzania joined the IMF in 1962, and started transactions with the Fund in the 1980s. When Tanzania accepted IMF’s financial support, the programs aimed at fiscal consolidation. These Stand-by Arrangements, or later SAPs, aimed at reducing inflation and the fiscal deficit, as well as tax reforms, wage bill ceilings, and strengthening the private sector [42]. In more recent years, the relationship between Tanzania and the IMF has returned to consultations under the PSI, with continued emphasis on fiscal consolidation. According to the most recent letter of intent, Tanzania did not meet the social spending target [43].

Tax revenue collection is rather low at 13% of GDP [43]. Tanzania aims at increasing the tax base through an expansion of VAT [44]. Moreover, Tanzania decided to lower income taxes for the wealthy, shifting the tax burden to the rest of the population [45]. The IMF welcomes the new VAT Act but emphasizes that ‘more needs to be done’ regarding streamlining exemptions and refund mechanisms and, similar to the Malawi program, suggests the country eliminate corporate income tax exemptions and holidays. It suggests as well that Tanzania introduces property taxes [46]. Although economic performance of Tanzania looks rather positive with a steady annual GDP growth of 7% in the last two decades, the IMF notices that recently the performance has been mixed and considerable risks remain.

The IMF welcomes Tanzania’s attempt to transition to an interest rate based monetary framework, and Tanzania’s progress towards this adheres to previous IMF recommendations. The IMF notes that further measures to increase public revenue are needed, such as expanding export opportunities and adhering to fiscal consolidation over the medium to longer terms [43]. In the latest Financial System Stability Assessment, it points to the possibility of privatizing commercial state-owned enterprises to be listed on the Dar es Salaam Stock Exchange Market [43].

In the last PSI consultation in 2016 the IMF acknowledged that higher fiscal deficits could be sustained for some time if simultaneously the debt distress is kept low [46]; its formulated target, however, was lower than usual at 3.25% of GDP (compared to formerly 4.2% of GDP). Tanzania targeted a budget deficit of close to 4% of GDP in 2017/18, and capital spending was planned at 10% of GDP. However, budgetary revenue projections led to concerns and development projects were delayed. The IMF still projected a shortfall and advised further expenditure cuts. In 2018/19 an even lower budget deficit of 2.5% of GDP was targeted [43]. In the 2016 PSI consultation, Tanzania committed to improving social services in order to reduce poverty. However, fiscal consolidation is recommended to reduce government’s financial needs, and the Fund explicitly invites Tanzania to *‘revisit fiscal priorities to ensure that critical infrastructure projects, particularly in the energy sector, are implemented’* ([44], p. 40).

## Discussion

The above findings clearly indicate that, in order to reach the levels of health investment required to realize the SDG3, countries would need levels of GDP growth that they have never before witnessed. Even if this highly unlikely event were to happen in a distinct future, our country analyses underline the fact that GDP growth is still no guarantee for an increase in government health spending or poverty reduction. However, it is true that LICs and L-MICs will need to expand their economic base (and in ways that do not jeopardize SDGs related to the physical environment, including climate change) and take the political decision to invest those gains in social spending, including health.

As reported by the United Nations, GDP growth has been volatile and far below the target of 7% set for the LDCs in SDG8.1. The average rate of growth in LDCs has even decreased from 3.5% in the period 2000–2004 to 2.3% in the period 2010–2016 [47]. In our analysis, over the last 10 years, we saw GDP per capita increases in Uganda and in Tanzania, while it has been volatile and not growing in Malawi. Meanwhile, government health spending per capita over this same period went up in Malawi and in Tanzania, but went down in Uganda. As well, Tanzania’s steady growth of 7% annually did not initially lead to any increase of public health spending, which only started to match GDP growth in the last 2 years. This underscores once more that an increase in GDP does not always lead to higher government health spending.

Under the influence of structural adjustment in the past, our focus countries (as many others) have focused their economic policies on lowering budget deficits through reducing public expenditure. This continuing emphasis on fiscal austerity directly or indirectly leads to reduced (or insufficiently increased) investments in health. Under SAPs, health spending was cut in many countries [48].

Our analysis of the latest IMF programs and policy advice in the three countries showed that fiscal consolidation was still a prominent part of all three. Adjustment measures considered by Uganda in the years 2010–2013 were related to wage bill cuts/caps, consumption (VAT) tax increases, and pension reform, with the wage bill cuts/caps leading to salary erosion among public healthcare providers [16]. Tanzania has followed advice regarding the reduction of subsidies for agricultural products, wage bill cuts/caps, and pension reforms [16]. In addition to these, the government also decided to increase consumption taxes and electricity prices [9]. In the case of Malawi, the IMF program strongly emphasized the need for tight fiscal policies, recommending spending reductions on agricultural and fuel subsidies, and limits on public sector wage increases. Our findings on wage bill cuts imply outcomes similar to those found in studies of IMF programs in Sierra Leone and Guinea, which called for wage bill freezes or reductions during and after the Ebola crisis, and which led to serious reductions in health worker to population ratio in Sierra Leone, as well as in nearby Ghana and Senegal [10]. Consistent with our findings, research by Eurodad on conditions attached to IMF loans in 26 country programs approved in the years 2016 or 2017 revealed that, contrary to what the IMF has been propagating, the majority were geared towards fiscal consolidation, including conditions to restrict spending and/or increase taxes [15].

With a view to increasing tax revenue, IMF advice focuses primarily on consumption taxes such as VAT (as in our three focus countries), which are generally regressive and hurt women and the poor disproportionately. Such taxes can contribute to or exacerbate existing poverty rates and (health) inequities. Analysis by the Commitment to Equity Institute revealed that in several of the twenty-nine countries they studied, including in Tanzania and Uganda, ‘*the extreme poverty headcount ratio is higher after taxes and transfers than before’* and identify consumption taxes as ‘*the main culprits of fiscally-induced impoverishment’* ([49] p. 4). In each of the three focus countries, recent IMF programs recommend an expansion of VAT. Furthermore, in Tanzania the government decided to lower income taxes for the wealthy and instead shifted the tax burden to the rest of the population [45]. More progressive tax advice does appear in IMF programs, as we have noted, but whether such measures are adopted by governments or are sufficient to improve substantially public revenues and subsequent increases in health and social protection spending remains moot. Corporate tax rates in all three countries, for example, have not increased over the past decade [50].Property taxes, as proposed by the IMF for Tanzania, may be progressive if applied only to large land-holdings of wealthier groups, but could also be regressive if affecting small-hold farmers or poorer urban dwellers.

Since 2010, IMF programs started to include non-binding social spending floors [13]. Although social spending floors are a move in the right direction, the targets would need to be set at a meaningful level to bring countries closer to achieving SDG3, which is not yet the case for our three focus countries. In Malawi the social spending target is too low to have any substantive health impact. In Uganda, the IMF advised the government to increase much needed social spending but only when economic growth recovered. In Tanzania the IMF advised the government to increase investments in the infrastructure sector while at the same time freezing total spending.

Another similarity found in all three countries is their adherence to the IMF’s advice of a floating exchange rate. In the case of currency devaluation this can drive up prices of imported goods important for health, including medical supplies and medication, and can rapidly harm the entire health care service provision of a country. Additionally, all three countries have adopted, or are in the process of adopting, an inflation-targeting framework, which is usually implemented through maintaining high interest rates. High interest rates can be harmful for the economy, by increasing the cost of borrowing for small and medium sized enterprises (reducing their expansion and employment creation) and for the government (increasing their debt burden and thereby reducing their fiscal space). In spite of the fact that there is no empirical consensus that inflation rates of up to 20% are harmful for the economy, the IMF recommends setting inflation targets at ‘lower single digits’ [51].

Clearly, alternative policies are needed to make greater progress towards not only SDG3, but other SDGs that have indirect but important impacts on health. For country level policies, experts have been proposing different options for more accommodating macroeconomic policy to expand government expenditure. An empirical study carried out in 2017 for the ILO on fiscal space for social protection in relation with the SDGs in 187 countries [45] showed that a 2% increase of a country’s fiscal deficit could result in vast increases in the resources available for public health. The authors suggest that.

it is important to carry out a rigorous assessment of fiscal sustainability within a country, taking into account not only economic aspects such as debt burden, revenue generation capacity and likely GDP growth trajectory but also the potential opportunity cost of foregoing social spending. ([43] p.49)

The second channel of a more accommodating macroeconomic policy is via more expansionary monetary policy. Low inflation, although still considered to be the best tool to ensure macroeconomic stability and growth, has become a goal in itself pushed for by the IMF [45, 52]. The views on what consists an ‘acceptable’ and ‘safe’ inflation level have been very diverse and conflicting, ranging from 3 to 40% [45, 52]. The most common tool to maintain low inflation is by setting high interest rates. If this policy was loosened and interest rates lowered, it would be less costly for both government and entrepreneurs to borrow and thus make investments, including in the public health sector.

These options need to be further explored at the country level. In addition, we question the use of a unique SDG target on GDP growth. It is known that both the reduction of poverty (SDG1) and a healthier population contribute to economic growth [2, 4], as does SDG4 (quality education) [53] and SDG10 (reduced inequalities) [2, 54]. The inclusion of SDG target 8.1 risks bringing more health harm than good, as it suggests that GDP growth is an end in itself. In doing so, it presents governments with the option to put more emphasis on SDG8.1 following the conventional, but empirically unfounded, argument that GDP growth will inevitably ‘trickle down’ and translate into a wealthier, healthier, and more inclusive society.

We do not deny that in order to increase spending on social sectors, including health, LICs and L-MICs will need to increase their overall public revenue. Current economic policies being pursued by, and/or promoted via IMF programs and policy advice, do not appear to result in significant GDP growth, nor lead to a sufficient level of investments in health, and DAH remains inadequate to meet the shortfalls. The funding gap is not so large, though, when compared to the income that is lost every year due to tax avoidance and tax evasion, to debt repayments, and to unfair trade arrangements [55]. Some, but not all, of these international challenges are targeted in SDG17 – a global partnership for sustainable development. SDG17 includes several targets aimed at increasing finance for development, including a call on high-income countries (HICs) to implement official development assistance (ODA) commitments, support developing countries to increase domestic resource mobilization, and reduce the level of debt service of developing countries. Progress on this SDG is conspicuously lagging behind. Commitments to increase ODA and improve its quality are not implemented, developing countries’ debt service payments are rising as percentage of their GDP, and the rate of taxation relative to GDP has fallen for Sub-Saharan Africa and for the LDCs [56, 57] What is missing in this SDG, is a target for reducing tax avoidance and evasion, even though global losses due to tax avoidance are estimated at USD 500 billion annually [58]. SDG16 does include a target on reducing illicit financial flows, but the SDG progress reports do not mention monitoring of this indicator [59].

Although SDG8.1 identifies its GDP growth goal for LDCs only, its legitimation of GDP as the most appropriate economic metric can influence its continued adherence in LICs, L-MICs and HICs. This will be problematic for health and development in the LDCs, since aggregate (global) GDP growth increases the already oversized carbon footprint of HICs, and to lesser extent LICs and L-MICs, and stretches the economy beyond the planet’s ecological ceiling [60]. Emphasis on the constant pursuit of GDP growth is also likely to prevent HICs from taking action towards the realization of SDG17.

Several alternative measurements to the GDP have been developed over the years. The Human Development Index (HDI), first introduced in 1990, measures achievements in three basic dimensions of human development—a long and healthy life, access to education, and a decent standard of living [61]. Building on that, the Human Development Report 2010 introduced the inequality-adjusted HDI (IHDI) [62]. The same year, the Global Multidimensional Poverty Index was developed. It is a measure of serious deprivations in the dimensions of health, education, and living standards that combines the number of deprived and the intensity of their deprivation. While it measures the same dimensions as the HDI, it has more indicators, which makes it more complicated to calculate but less susceptible to bias [62]. GDP per capita and HDI have similar trajectories according to trend data for the focus countries of this study. However, in all three countries, there is a loss in the HDI figures when adjusted to inequality. The loss stands at approximately 30% for Malawi, 28% for Uganda, and 25% for Tanzania [61]. This fact is contradictory with the neoliberal suggestion that constantly increasing economic growth will finally eliminate inequalities, as once depicted by the iconic Kuznets’ curve [60].

These indicators are already widely used alongside, but not replacing, the GDP. An alternative that could replace GDP as a policy goal is the Genuine Progress Indicator (GPI). GPI has already been used by some states of the United States of America, with Costa Rica, Scotland, and Sweden soon to follow. The GPI starts with a measurement of GDP but then takes into account positive non-monetary factors such as household and volunteer work, and subtracts negative factors such as pollution, resource depletion and crime. It also adjusts for inequality. If governments shifted towards pursuing a maximization of the GPI instead of the GDP, they would adopt policies that would facilitate inclusive and sustainable economic outcomes, accelerate progress towards social well-being and allow for a fairer distribution of wealth and health across the globe [63, 64]. As suggested by Raworth, economic impact assessments should be based on indicators of ecological overshoot and domestic social inclusion in order to achieve ‘*human prosperity in a flourishing web of life’* ([60] p. 60).

### Limitations

As we conducted a purposeful selection of the most recent IMF documents for analysis, our approach was not exhaustive and might lack relevant literature that would have given a deeper insight. In addition, we chose to focus primarily on the IMF’s role in the countries’ policy-making acknowledging its prominent role in macroeconomic advice. However, to expand the scope of knowledge on the full picture of macroeconomic development in the three focus countries, other influential international financial institutions and organizations, such as the World Bank, regional development banks, and multi/bilateral donors could have been taken into account. This study focused on three countries in the East African region, which diminishes generalizability and external validity of the study. However, as our focus countries share political and economic features with several LICs in Africa, the insights gained may give rise to further studies and evidence-based advocacy in the region.

## Conclusion

Our desk-based analysis of three East African focus countries affirmed findings of other studies, showing that GDP increase does not automatically translate to an increase of health spending, partly a result of IMF structural adjustment programs. Although the IMF has started to acknowledge the importance of social development, its policy advice is still adhering to austerity and pro-cyclical economic development with potentially harmful effects on a country’s population health. In order to increase chances to achieve the SDGs, notably SDG3, the international community should abandon SDG8.1, choose alternative indicators to measure economic development and put emphasis on SDG17.

We accept that this policy advice is provisional, as it is based on analyses of extant studies and several different databases. Our provisional advice could be better informed with follow-up stakeholder interviews, as the opinion and input of those with considerable knowledge of policy concerns within each of our focus countries, and across LDCs more generally, could substantiate and/or elaborate on our own findings and conclusions. We encourage such work to be undertaken, as the countdown on Agenda 2030 continues.

## Data Availability

The datasets used and analyzed during the current study are available from the corresponding author on reasonable request.

## Abbreviations

DAH: Development Assistance for Health
ECF: Extended Credit Facility
GDP: Gross Domestic Product
GGHE-D: Domestic General Government Health Expenditure
GPI: Genuine Progress Indicator
HDI: Human Development Index
HICs: High-Income Countries
HLPF: High Level Political Forum
IHDI: Inequality-adjusted Human Development Index
ILO: International Labor Organization
IMF: International Monetary Fund
LDCs: Least Developed Countries
LICs: Low-Income Countries
L-MICs: Lower Middle-Income Countries
MDGs: Millennium Development Goals
ODA: Official Development Assistance
OECD: Organization for Economic Co-operation and Development
PSI: Policy Support Instrument
SAPs: Structural Adjustment Programs
SDGs: Sustainable Development Goals
UHC: Universal Health Coverage
UNHEEG: United Nations High-Level Commission on Health Employment and Economic Growth
USD: United States Dollar
VAT: Value-Added Tax
WHO: World Health Organization
